# Proline is required for male gametophyte development in Arabidopsis

**DOI:** 10.1186/1471-2229-12-236

**Published:** 2012-12-12

**Authors:** Roberto Mattioli, Marco Biancucci, Chiara Lonoce, Paolo Costantino, Maurizio Trovato

**Affiliations:** 1 Dipartimento di Biologia e Biotecnologie, Università di Roma La Sapienza, P.le Aldo Moro 5, Rome, 00185, Italy

**Keywords:** Proline, Male gametophyte, *Arabidopsis*, *p5cs1 p5cs2/P5CS2*

## Abstract

**Background:**

In crosses between the proline-deficient mutant homozygous for *p5cs1* and heterozygous for *p5cs2 (p5cs1 p5cs2/P5CS2)*, used as male, and different Arabidopsis mutants, used as females, the *p5cs2* mutant allele was rarely transmitted to the outcrossed progeny, suggesting that the fertility of the male gametophyte carrying mutations in both *P5CS1* and *P5CS2* is severely compromised.

**Results:**

To confirm the fertility defects of pollen from *p5cs1 p5cs2/P5CS2* mutants, transmission of mutant alleles through pollen was tested in two ways. First, the number of progeny inheriting a dominant sulfadiazine resistance marker linked to *p5cs2* was determined. Second, the number of *p5cs2/p5cs2* embryos was determined. A ratio of resistant to susceptible plantlets close to 50%, and the absence of aborted embryos were consistent with the hypothesis that the male gametophyte carrying both *p5cs1* and *p5cs2* alleles is rarely transmitted to the offspring. In addition, in reciprocal crosses with wild type, about 50% of the *p5cs2* mutant alleles were transmitted to the sporophytic generation when *p5cs1 p5cs2/P5CS2* was used as a female, while less than 1% of the *p5cs2* alleles could be transmitted to the outcrossed progeny when *p5cs1 p5cs2/P5CS2* was used as a male. Morphological and functional analysis of mutant pollen revealed a population of small, degenerated, and unviable pollen grains, indicating that the mutant homozygous for *p5cs1* and heterozygous for *p5cs2* is impaired in pollen development, and suggesting a role for proline in male gametophyte development. Consistent with these findings, we found that pollen from *p5cs1* homozygous mutants, display defects similar to, but less pronounced than pollen from *p5cs1 p5cs2/P5CS2* mutants. Finally, we show that pollen from *p5cs1 p5cs2/P5CS2* plants contains less proline than wild type and that exogenous proline supplied from the beginning of another development can partially complement both morphological and functional pollen defects.

**Conclusions:**

Our data show that the development of the male gametophyte carrying mutations in both *P5CS1* and *P5CS2* is severely compromised, and indicate that proline is required for pollen development and transmission.

## Background

In addition to its role as proteinogenic amino acid, and as a molecule involved in responses to a number of biotic and abiotic stresses, proline has been implicated in plant development, particularly flowering and reproduction [1-3]. The first convincing evidence that proline may play a key role in plant reproduction under normal unstressed conditions, came from measurements of free proline content in a number of plant species, which revealed low levels of proline in vegetative tissues followed, after flower transition, by proline accumulation in reproductive tissues and organs [4-8]. Chiang and Dandekar [7], for example, reported that free proline accumulates in *Arabidopsis* reproductive tissues up to 26% of the total amino acid pool, while in vegetative tissues represents only 1-3%. Among floral organs, different authors [7-11] pointed out that the floral organ with the highest proline content is pollen, where proline may represent more than 70% of the total amino acid content [8].

It is not clear, to date, the reason for such a massive proline accumulation in pollen. Because pollen grains undergo a process of natural dehydration, a role of compatible osmolyte capable of protecting cellular structures from denaturation, has been proposed by some authors [7,12,13], while others [14] have postulated a role for proline as a source of energy or as metabolic precursor to support the rapid and energy-demanding elongation of the pollen tube. On the other hand, the rapid elongation of the pollen tube requires extensive synthesis of cell wall proteins [15], some of which are rich in proline or hydroxyproline stretches, and proline accumulation may be needed to sustain the synthesis, at high levels, of proline-rich cell wall proteins [16].

Irrespective of its function, proline may accumulate in pollen due to an increased transport from external sources, or to an increased ratio between synthesis and degradation of endogenous proline, or because of a combination of the two, but no conclusive evidence has been produced, as yet, to distinguish among these alternative models. Long distance transport of proline through phloem vessels has been documented [17,18] and since *AtProT1* (AT2G39890), a gene encoding an amino acid carrier recently shown to mediate proline uptake in plants, is highly expressed in mature pollen [19], transport has been proposed to account for proline accumulation in pollen grains. However single, double, and triple knockout mutants for all the genes belonging to the AtProT family are available, and none of them show difference, compared to wild type, neither in proline content, nor in pollen germination efficiency [19], raising the possibility that endogenous proline synthesis may be responsible for, or contribute to proline accumulation in pollen.

In higher plants proline synthesis proceeds from glutamate that is converted to proline in a two-step pathway catalyzed by the enzymes Δ^1^-pyrroline-5-carboxylate ynthetase (P5CS), and Δ^1^-pyrroline-5-carboxylate reductase (P5CR). The existence of an alternative route for proline synthesis, converting ornithine to proline by the action of δ-ornithine-amino-transferase (δ-OAT; At5g46180) and P5CR, has been hypothesized by some authors [20,21]. However the relevance of this pathway for proline synthesis has been recently questioned [22] and glutamate may be the only precursor of proline synthesis in plants. P5CS, regarded as the rate-limiting enzyme for proline biosynthesis in plants, is encoded in Arabidopsis by the two paralog genes *P5CS1* (At2g39800) and *P5CS2* (At3g55610) [23], while no paralog genes have been described for *P5CR* (At5g14800).

T-DNA insertional mutants have been characterized [1,3,24] for both *P5CS1* (SALK_063517, *p5cs1-4*) and *P5CS2* (GABI_452G01, *p5cs2-1*;FLAG_139H07, *p5cs2-2*), providing hints for assigning gene functions. *P5CS1* is responsible for abiotic stress-induced proline accumulation, as homozygous *p5cs1* mutants do not accumulate proline upon stress induction and are hypersensitive to environmental stresses [3,24], while *P5CS2* is necessary for embryo development, as homozygous *p5cs2* mutants are embryo lethal and the *p5cs2* mutant allele can be propagated only in *p5cs2*/*P5CS2* heterozygous mutants [1,24]. In addition, both genes have been shown to modulate flower transition in Arabidopsis, as the flowering time of mutants homozygous for *p5cs1* and heterozygous for *p5cs2*, is more delayed than that of the single *p5cs1* mutant [1,2].

In the course of a genetic screen designed to identify the floral pathway(s) proline interacts with in Arabidopsis (Mattioli et al., in preparation), we found that the *p5cs2* mutation was rarely transmitted to the offspring when the proline-deficient *p5cs1 p5cs2/P5CS2* was used as a pollen donor suggesting yet another role for proline in affecting male fertility. This prompted the analysis presented in this work, aimed to evaluate the role of endogenous proline in pollen development and fertility. We show here that the development of the male gametophyte carrying mutations in both *P5CS1* and *P5CS2* is severely compromised, indicating a role for proline in pollen function and development.

## Results

In crosses between *p5cs1 p5cs2/P5CS2*, used as male, and Arabidopsis flowering time mutants, used as females, aimed to understand the flowering pathway proline interacts with (Mattioli et al., in preparation), the *p5cs1* mutant allele was always transmitted to the outcrossed progeny, while the transmission frequency of the *p5cs2* mutant allele was exceedingly low (in average 0.8 ± 0.1%). Since no obvious gametophytic defects have been ever noticed neither on *p5cs1* nor on *p5cs2* single mutants, this result suggests that a male fertility defect may be linked to pollen grains bearing mutations in both *P5CS1* and *P5CS2* genes.

### Segregation of the *p5cs2* mutant allele in a seed population from self-pollinated *p5cs1 p5cs2/P5CS2* plants is consistent with the presence of a gametophytic mutation

**Figure 1:**
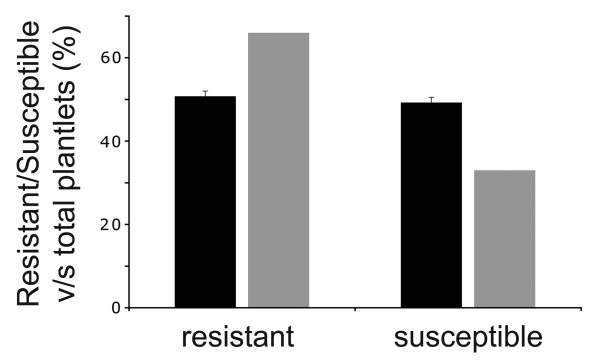
**Segregation of the *p5cs2 *mutant allele in a self-pollinated *p5cs1 p5cs2/P5CS2 *population.** Percentage of resistant versus total plantlets (black left column) or susceptible versus total plantlets (black right column) grown under sulfadiazine selection are shown. The corresponding gray columns represent the percentages expected if the *p5cs1 p5cs2/P5CS2* were not a gametophytic mutant. Values represent the means of six independent experiments ± SE.

To verify the fertility defects of pollen from *p5cs1 p5cs2/P5CS2* mutants, the segregation of the sulfadiazine gene – a dominant resistance marker associated to the T-DNA insertion on *P5CS2* – was analyzed in a seed population from selfed *p5cs1 p5cs2/P5CS2* plants*.* Since the homozygous *p5cs2* single mutant is embryo lethal [1], we expected that the segregation of the *p5cs2*-linked sulfadiazine resistance in the selfed population would approximate a 2:1 ratio of resistant over susceptible plants. If, on the other hand, the segregation ratio for the *p5cs2* mutant allele should approximate a 1:1 ratio, a fertility defect for the gametophyte carrying both *p5cs1* and *p5cs2* mutations would be confirmed. To clarify this point, 831 seeds, from *p5cs1 p5cs2/P5CS2* plants, were planted on sulfadiazine plates in six independent experiments. As shown in Figure 1, the *p5cs2* mutation segregated in a 1:1 resistant:susceptible ratio, as about 50% (50.6 ± 0.1%; n= of 831; p*** < 0.000 1; χ^2^ = 45) plantlets were sulfadiazine resistant (black bars in Figure 1), consistent with the hypothesis that the *p5cs1 p5cs2* gametophyte is infertile and can hardly transmit the *p5cs2* allele to the sporophytic generation.

### The absence of aborted embryos in *p5cs1 p5cs2/P5CS2* siliques is consistent with a gametophytic mutation hampering homozygous formation

**Figure 2:**
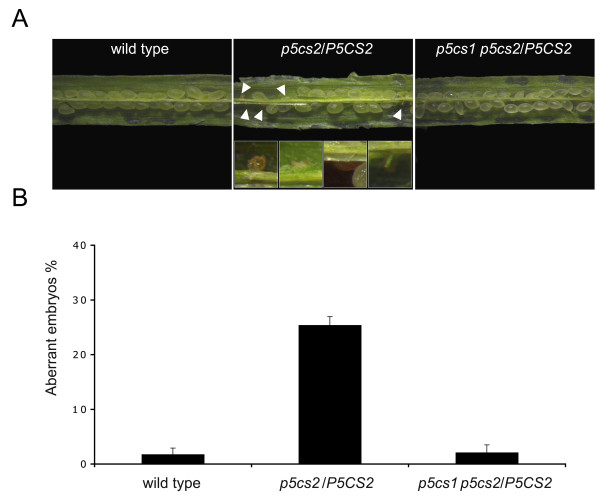
**Morphological analysis of seed defects in siliques of wild type, *p5cs2/P5CS2 *and *p5cs1 p5cs2/P5CS2 *plants.** The percentage of aberrant versus normal seeds was scored in wild type, *p5cs2/P5CS2* heterozygous and *p5cs1 p5cs2/P5CS2* mutants. While siliques from heterozygous *p5cs2* mutant (**A** and **B**, middle) present ~25% of aberrant seeds (white arrowheads) the percentage of seed abortion of *p5cs1 p5cs2/P5CS2* siliques (**A** and **B**, right) is indistinguishable from wild type (**A** and **B**, left). Details, at higher magnification, of some of the aberrant seeds of figure B (middle) are shown in the insets. Values represent the means of four independent experiments ± SE.

To further confirm the gametophytic defect of the *p5cs1 p5cs2/P5CS2* mutant, the incidence of embryo abortion was examined in the siliques of *p5cs1 p5cs2/P5CS2*. Aborted seeds could be observed in the siliques of a self-pollinated heterozygous *p5cs2*/*P5CS2* mutant, accounting for about 25% of the total seeds and corresponding to the genotype *p5cs2/p5cs2* (Figure 2, middle, see white arrowheads) compared with about 1% of spontaneous seed abortion found in wild type siliques (Figure 2, left). However, in case a gametophytic defect should impede homozygous formation, the absence of aborted embryos in the siliques of *p5cs1 p5cs2/P5CS2* would be expected. Indeed, as shown in the right part of Figure 2, *p5cs1 p5cs2/P5CS2* siliques show a wild type-like phenotype with less than 1% aborted seeds. These data are in agreement with the hypothesis that the (male) gametophyte carrying both *p5cs1* and *p5cs2* alleles is rarely transmitted to the offspring.

### Reciprocal crosses between *p5cs1 p5cs2/P5CS2* and wild type confirm the male gametophytic defects associated to pollen grains mutated in both *P5CS1 and P5CS2*

To confirm genetically the male sterility of pollen grains carrying both *p5cs1* and *p5cs2* mutant alleles, reciprocal backcrosses were made between *p5cs1 p5cs2/P5CS2* and wild type plants. All seeds produced by the outcrossed siliques were collected and germinated on sulfadiazine-containing media, to follow the transmission of the *p5cs2* mutant allele (Table 1). As shown in Table 1, when pollen from *p5cs1 p5cs2/P5CS2* plants was used to fertilize wild type pistils*,* the *p5cs2* mutant allele could be transmitted to the progeny only in 1 out of 132 plants (0.76 ± 0.09%; χ^2^=128; P<0.001), producing a *p5cs1 p5cs2/P5CS2* genotype. In contrast, transmission of the *p5cs2* allele in reciprocal crosses occurred in 41 out of 92 cases, suggesting that mutations in both *P5CS1* and *P5CS2* have no effects on the female gametophyte.

**Table 1:** **Reciprocal crosses between*p5cs1 p5cs2/P5CS2*mutants and wild type plants**

**Crosses (Female*×*male)**	**Genotype of the progeny**	
	***p5cs1 p5cs2/P5CS2 p5cs1 p5cs2/P5CS2***	
**wt***×****p5cs1 p5cs2/P5CS2***	1/132 (0.75%)***	131/132 (99.15 %)***
***p5cs1 p5cs2/P5CS2****×***wt**	41/92 (45%)	51/92 (55%)

Reciprocal crosses between mutants homozygous for *p5cs1* and heterozygous *for p5cs2* and wild type plants. *** Significantly different from the expected segregation ratio of 50% (P < 0.001).

**Figure 3:**
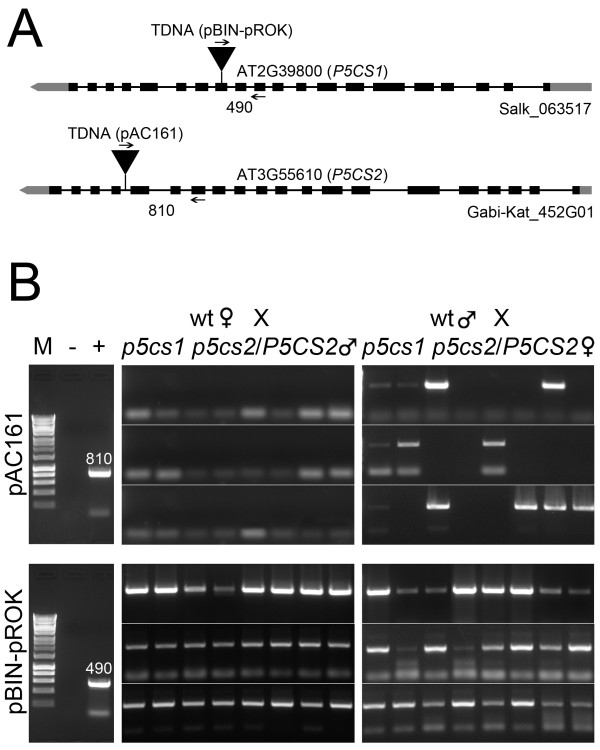
**Molecular analysis of reciprocal crosses between *p5cs1 p5cs2/P5CS2 *mutants and wild type.** (**A**) Schematic drawing of the insertional mutant *p5cs1*(Salk_063517) and *p5cs2* (Gabi-Kat _452G01). The position of the T-DNA insertion, the location of the PCR primers used for genotyping, and the expected length of the PCR products are shown for *P5CS1* (At2G39800), and *P5CS2* (At3G55610). (**B**) PCR amplification of T-DNA insertions associated to *P5CS2* (PAC161 T-DNA, top panels) and *P5CS1* (pROK T-DNA, bottom panels) in reciprocal crosses between *p5cs1 p5cs2/P5CS2* and wild type. Samples from 24 randomly chosen outcrossed plantlets are shown. The Results shown on top panels indicate that the *P5CS2* mutation is never transmitted to the progeny (0/24) when *p5cs1 p5cs2/P5CS2* serves as a male (top panel left), but is normally segregated (12/24), when serves as a female. Negative and positive controls are shown in the leftmost panel, relative to the amplification of wt (− ,top and bottom leftmost panel), *p5cs2/P5CS2* (+, top leftmost panel) and *p5cs1* (+, bottom leftmost panel) parental genotypes. The numbers next to the PCR products represent the expected molecular weight expressed in Kb for the *P5CS2*-T-DNA (top panel), and *P5CS1*-T-DNA (bottom panel) junction fragments, respectively.

To confirm these data at molecular level, 24 individuals were randomly chosen from each outcrossed progeny (male *p5cs1 p5cs2/P5CS2 ×* female wt, and female *p5cs1 p5cs2/P5CS2 ×* male wt) and analyzed by PCR for the presence of the T-DNA insertion on *P5CS2* (Figure 3A and 3B, top panel), and on *P5CS1* (Figure 3A and 3B, bottom panel). As shown in the top panel of Figure 3B, when *p5cs1 p5cs2/P5CS2* was used as a male, a combination of primers specific for *P5CS2* and for the T-DNA vector pAC161 could not detect the presence of a T-DNA insertion on *P5CS2* (Figure 3B leftmost top panel). On the contrary, when *p5cs1 p5cs2/P5CS2* was used as a female, a PCR product specific for the T-DNA insertion on *P5CS2* was amplified in 12 out of 24 samples (Figure 3B rightmost top panel), providing molecular support that the transmission of the *p5cs2* mutant allele is compromised in male, but not in female gametophytes from *p5cs1 p5cs2/P5CS2* plants. In addition, as shown in the bottom panel of Figure 3B, all the samples analyzed detected both the presence of a mutant *p5cs1* allele (Figure 3B left and right bottom panel), by using a couple of primers specific for *P5CS1* and for the T-DNA vector pROCK, and the presence of a wild type *P5CS1* allele (not shown), by using a primer pair specific for *P5CS1*, indicating that the chosen plants derived from an outcrossing event, and excluding the possibility of an unintended contamination from self-pollinated parental genotypes. Overall, the reciprocal crosses with wild type plants provide genetic and molecular evidence that *p5cs1 p5cs2/P5CS2* is impaired in male fertilization*.*

### Morphological and functional analysis of pollen from *p5cs1 p5cs2/P5CS2* mutants reveals severe defects in pollen development

**Figure 4:**
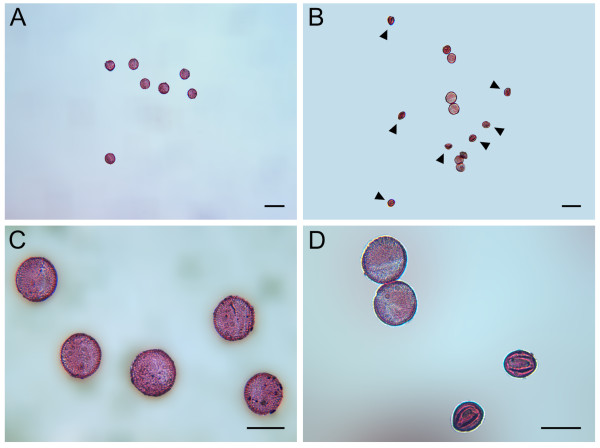
**Morphological analysis of pollen from *p5cs1 p5cs2/P5CS2 *and wild type.** Acetic orcein stain of pollen from wild type (**A** and **C**) and *p5cs1 p5cs2/P5CS2* plants (**B** and **D**) at two different magnifications show the presence in the latter pollen of small and shriveled pollen grains (arrows) alongside normal-looking grains. Bars = 50 μm (A, B) and 25 μm (C,D).

**Figure 5:**
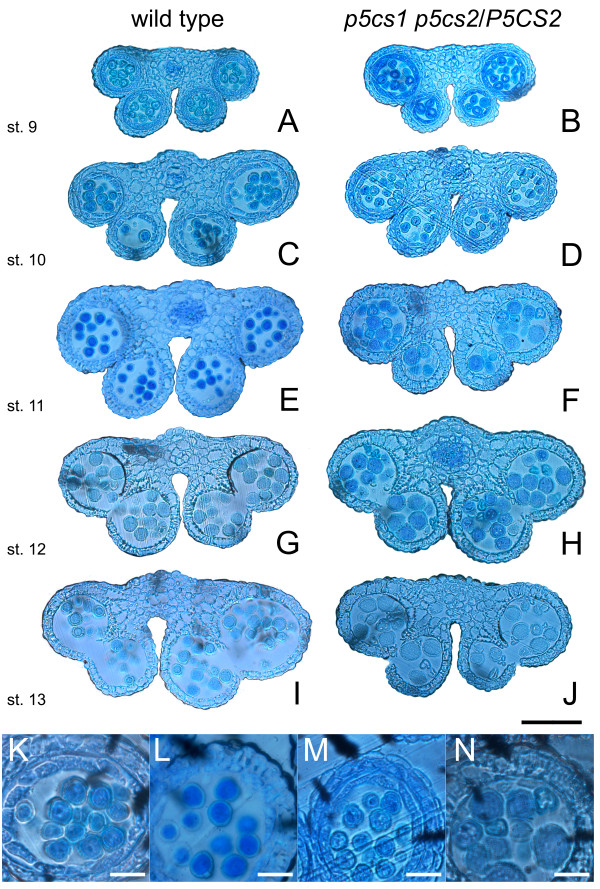
**Histological analysis of wild type and mutant pollen.** Cross-sections of anthers of different developmental stages. Toluidine blue-stained cross-sections of anthers from wild type (**A**,**C**,**E**,**G**,**I**) and *p5cs1 p5cs2/P5CS2* (**B**,**D**,**F**,**H**,**J**) from stage 9 to 13 are shown. The first clear differences between *p5cs1 p5cs2/P5CS2* and wild type anthers appear from stage 11 (E,F), when two populations of pollen grains can be distinguished in the pollen sacs. Bar = 100 μm. (**K**-**N**) Details at higher magnification of anthers from wild type (K,L) and *p5cs1 p5cs2/P5CS2* (M,N), relative to stage 10 (K,M) and 11 (L,N), respectively. Small misshaped pollen grains are clearly visible from stage 11 (E). No significant alterations, compared to wild type, are seen in anthers from *p5cs1 p5cs2/P5CS2* before stage 11. Bar = 25 μm.

**Figure 6:**
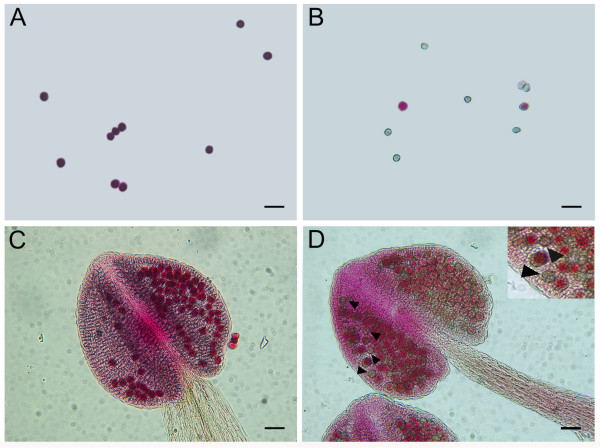
**Alexander's stain of pollen from wild type and *p5cs1 p5cs2/P5CS2 *plants.** Alexander's staining of pollen and anthers from wild type (**A**,**C**) compared to *p5cs1 p5cs2/P5CS2* mutant (**B**,**D**) reveals that a fraction of the mutant pollen population, looking small and misshaped, is not viable, as does not assume Alexander's stain. In contrast the remaining fraction of the pollen population appears as red as, and indistinguishable from wild type pollen. A detail of pollen grains from *p5cs1 p5cs2/P5CS2* mutant is shown in the inset at higher magnification. Bars = 50 μm.

**Figure 7:**
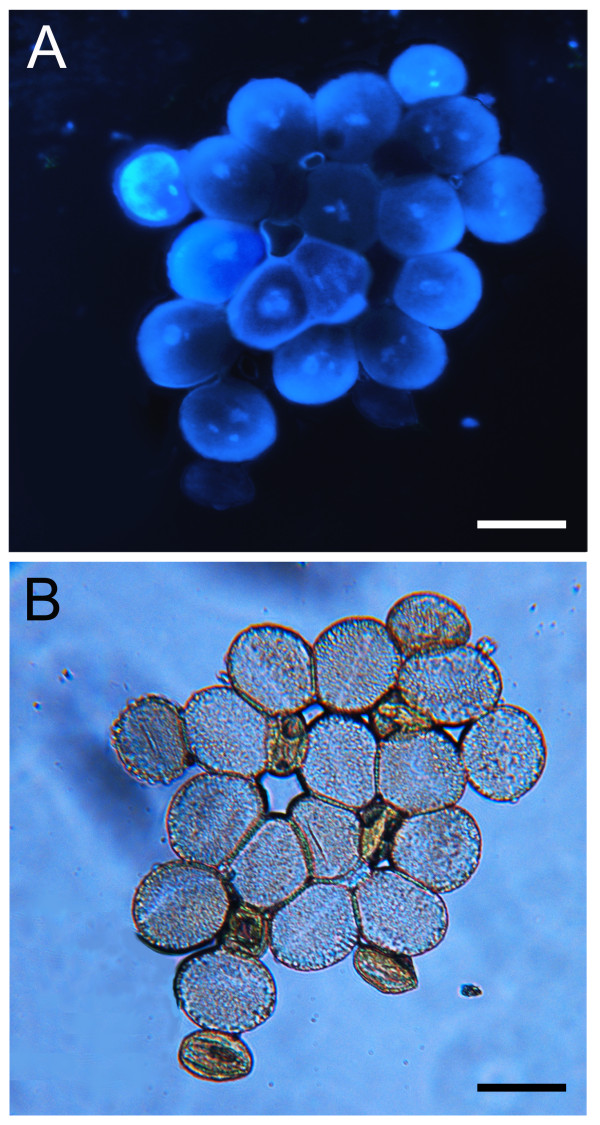
**DAPI analysis of pollen from wild type and *p5cs1 p5cs2/P5CS2.*** (**A**) DAPI staining of mature pollen from *p5cs1 p5cs2/P5CS2*. The small misshaped pollen of the mutant pollen population appears highly degenerated and depleted of nucleus. In contrast the larger and wild-type looking pollen grains display up to three nuclei, as in normal wild type pollen. (**B**) Bright-field image of the same picture showing large and small mutant pollen grains. Bar = 25 μm.

**Figure 8:**
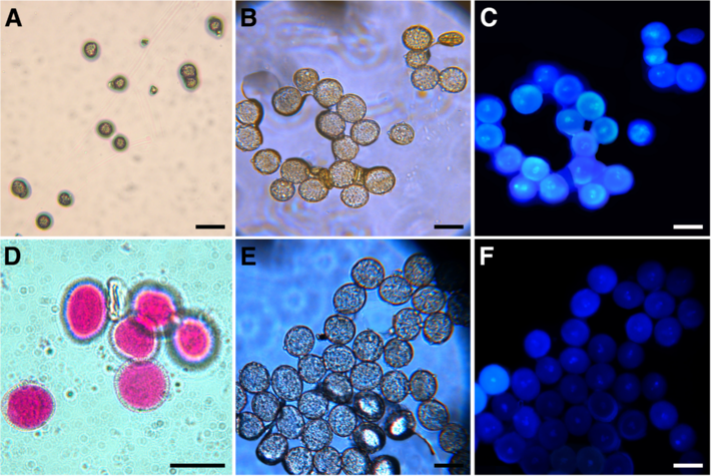
**PCR analysis of large and small pollen grains from *p5cs1 p5cs2/P5CS2 *plants.** DNA extracted from pools of large and small pollen grains from *p5cs1 p5cs2/P5CS2* plants was genotyped by PCR for the presence or absence of insertional mutations in *P5CS1* and *P5CS2.* No DNA could be amplified from the small pollen population (bottom panel), while both *p5cs1* and *p5cs2* mutant alleles, as well as the wild type *P5CS2* allele, were amplified from large pollens (top panel).

**Figure 9:**
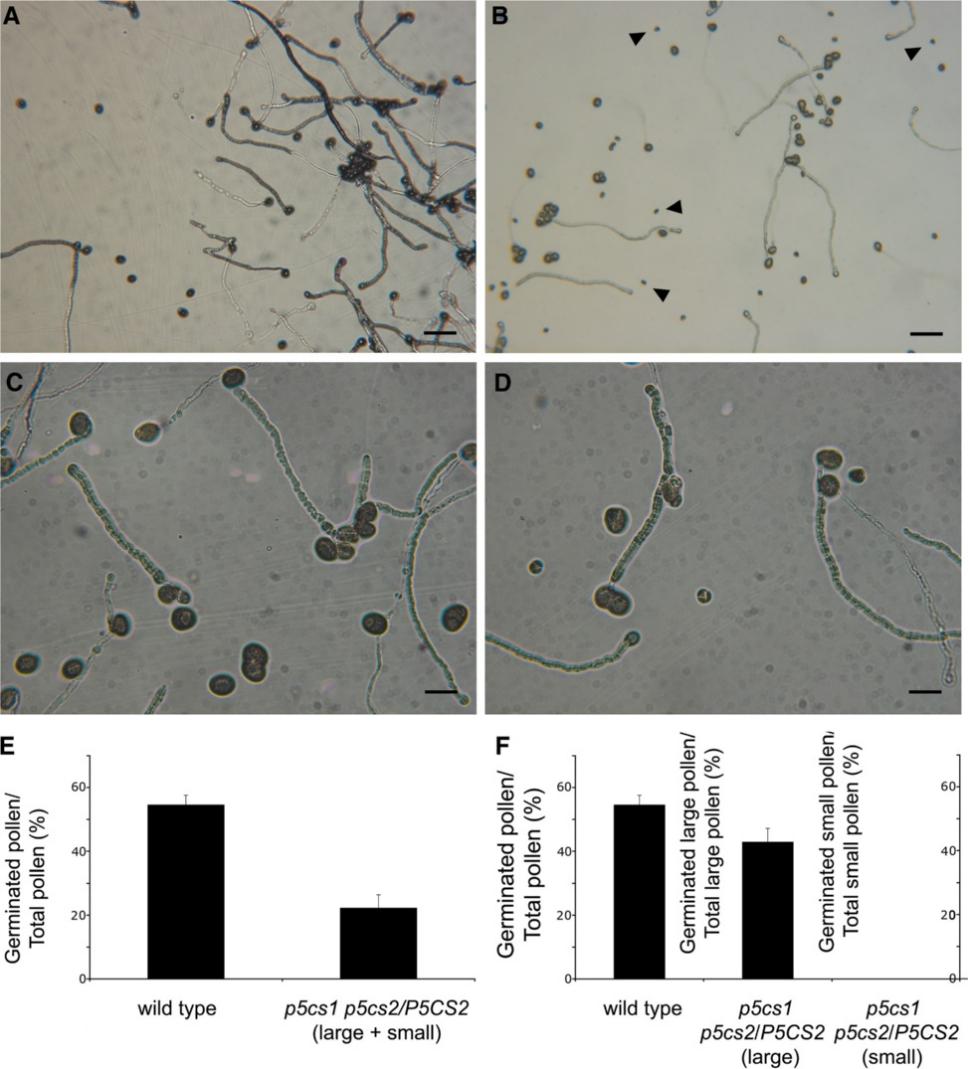
**Morphological analysis of pollen from *p5cs1 *homozygous mutants.** In support of a proline requirement for pollen development, morphologic analysis of pollen from *p5cs1* homozygous mutants revealed the presence of degenerated pollen grains (**A**-**D**), unstained with Alexander's stain (D) and lacking visible nuclei with DAPI staining (C, and bright-field control in B). Up to three nuclei are visible in pollen from wild type control (**F**, and bright-field control in **E**). Bars= 50 μm (A), 25 μm (B-D).

If *p5cs1 p5cs2* pollen is impaired in male fertilization, morphologic abnormalities in pollen grains may be expected. Indeed, as shown in Figure 4, microscopic analysis of mature pollen from *p5cs1 p5cs2/P5CS2* plants, stained with acetic orcein, revealed (Figure 4B and D), alongside normal-looking grains, a population of small, misshaped and shriveled pollen grains, roughly accounting for half of the total pollen population (46.23 ± 1.2%). This evidence indicates that in plants homozygous for *p5cs1* and heterozygous for *p5cs2*, the development of the male gametophyte is impaired and suggests that this defect may arise in pollen of *p5cs1 p5cs2* genotype. To understand at which stage of pollen development the aberrations took place, toluidine-stained histological cross-sections of *p5cs1 p5cs2/P5CS2* anthers, from different developmental stages [25], were prepared and analyzed in comparison to wild type. As shown in Figure 5, the first clear differences between *p5cs1 p5cs2/P5CS2* and wild type anthers appear from stage 11 (Figure 5F and N), when two populations of pollen grains – one similar to wild type, and another showing smaller size and initial signs of degeneration - can be distinguished in the pollen sacs. In order to assess whether this small abnormal pollen is vital, pollen grains from wild type and *p5cs1 p5cs2/P5CS2* mutants were treated with Alexander’s stain, a staining procedure capable of distinguishing viable, red-colored pollen from non viable, green or unstained pollen [26,27]. As shown in Figure 6, a population of small and abnormal pollen grains, representing 47.5 ± 2.1% of the total pollen grains, appear selectively unstained when treated with Alexander’s stain (Figure 6B and D, and Additional file 1: Figure S1). In contrast, the larger and normal-looking pollen present in the pollen population from *p5cs1 p5cs2/P5CS2* plants, takes up Alexander’s stain and appear as red as wild type pollen (Figure 6A and C, and Additional file 1: Figure S1). In addition, within the pollen population from *p5cs1 p5cs2/P5CS2* mutants*,* while the large pollen grains seem to have a normal nuclear content, the small and unviable pollen grains appear degenerated and devoid of DNA as judged by DAPI (Figure 7A and B) and PCR analysis (Figure 8). Indeed, in DAPI-stained pollen grains from *p5cs1 p5cs2/P5CS2*, no nuclei were ever observed in the small and abortive pollen grains, while up to three nuclei can be seen in the large and normal-looking pollen grains, as in normal pollen (Figure 9F).

### *In vitro* germination assays confirm that *p5cs1 p5cs2* pollen is essentially non viable, and suggest a quantitative role for proline in pollen development

**Figure 10:**
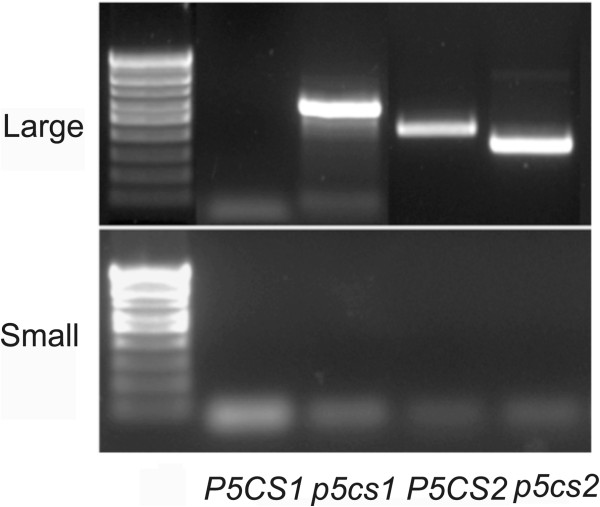
***In vitro *germination assays of pollen from *p5cs1 p5cs2/P5CS2 *plants.** To assess the viability of pollen from *p5cs1 p5cs2/P5CS2* plants, pollen from wild type (**A** and, at higher magnification, **C**) and *p5cs1 p5cs2/P5CS2* (**B** and, at higher magnification, **D**) was incubated *in vitro* on germination medium and scored for successful germination (pollen tube fully or partially elongated). In panel (**E**) the percentage of germinated versus total pollen is shown for wild type (left column) and mutant pollen (right column). In (**F**) the percentage of germination over total pollen is given for either large (middle column) or small (rightmost column) mutant pollen, compared to wild type (leftmost column). Bars = 100 μm (A, B) and 25 μm (C,D). Values in (E) and (F) represent the means of four independent experiments ± SE.

To further investigate the pollen viability of *p5cs1 p5cs2/P5CS2* plants, an *in vitro* germination assay was performed to test the capability of pollen from *p5cs1 p5cs2/P5CS2* plants to germinate *in vitro* (Figure 10B, D), as compared as to wild type pollen (Figure 10A, C). As reported in Figure 10, when pollen from *p5cs1 p5cs2/P5CS2* plants was grown on solid germination medium, the percentage of germinated versus total pollen (germinated + non germinated), including both large and small pollen grains, was reduced by ~ 50% (22 ± 4% compared to 54 ± 2.9% of control) compared to wild type (Figure 10, panel E). The fact that the germination percentage of wild type pollen was, on average, only 54 ± 2.9% can be accounted for by the relative inefficiency of this kind of experiments. It is known that *in vitro* germination cannot fully substitute for *in vivo* germination, with germination percentages showing ample variations [28]. In order to minimize this inherent variability, all experiments were carried out by placing, on the same microscope slides, pollen from wild type controls close to pollen from *p5cs1 p5cs2/P5CS2* mutants. Since genetic analysis indicated that half of the pollen grains from *p5cs1 p5cs2/P5CS2* plants are of *p5cs1 p5cs2* genotype, and morphological observations showed that ~ 50% of the pollen grains from *p5cs1 p5cs2/P5CS2* plants are small and misshaped, it is likely to hypothesize that all the aberrant pollens are *p5cs1 p5cs2.* According to this notion when large and small pollen grains are scored separately, all the small and degenerate pollen grains (*p5cs1 p5cs2* genotype) should fail to germinate, while all the large and wild type-looking pollens (*p5cs1 P5CS2* genotype) should elongate a pollen tube. To verify this point, the percentage of pollen germination was calculated separately for large and small pollen grains, either as number of large germinated pollen grains out of total large pollen grains, or as small germinated pollen grains out of total small pollen grains (Figure 10, panel F). Unexpectedly, while small pollen grains were never observed to germinate (Figure 10, panel B and D), the germination percentage of the large pollen grains vs. total large pollen grains from *p5cs1 p5cs2/P5CS2* plants was not 100%, but accounted for only 89% of that of wild type (43 ± 4.2% compared to 54 ± 2.9% of control pollen), as shown in Figure 10F, middle column, suggesting that the two pollen populations may not be pure, i.e. some pollen of *p5cs1 p5cs2* genotype might occasionally have degenerated and looking small, while some pollen of *p5cs1 p5cs2* genotype may be not, or not completely, degenerated and yet looking large. To clarify this point, pools of either large or small pollen grains were analyzed by PCR for the presence or absence of T-DNA insertions in *P5CS1* and *P5CS2.* As shown in Figure 8, no DNA could be amplified from the small pollen population, as expected from their morphological and functional degeneration and apparent absence of intact nuclei. When DNA from the large pollen population was examined for the presence of mutations in *P5CS1*, only the T-DNA insertion on *P5CS1* could be amplified (Figure 8), confirming that the large pollen grains are vital and indicating that the whole population contains the mutated *p5cs1* allele, as expected from a line homozygous for the *p5cs1* allele. However, when DNA from the large pollen population was examined for the presence of mutations in *P5CS2,* both wild type and mutant *P5CS2* allele were found, as witnessed by the presence of specific PCR products for both wild type and mutant *P5CS2* gene (see the two rightmost lines of Figure 8), confirming that the wild-type like pollen contains, to some extent, pollens carrying mutations in both *P5CS1* and *P5CS2*. The presence of pollens containing mutations in both *P5CS1* and *P5CS2* within the large, wild-type looking pollen population, suggests that pollen with genotype *p5cs1 P5CS2* is likely present within the degenerated pollen population. The simplest interpretation of these results is that proper development of the male gametophyte is dependent from proline availability, and that pollens of genotype *p5cs1 p5cs2* may contribute to some extent to the overall fertility defect of pollen from *p5cs1 p5cs2/P5CS2* mutants. An important implication of this reasoning is that pollen defects similar to, but less severe than those found on pollen grains from *p5cs1 p5cs2/P5CS2* plants should be present in pollen grains from homozygous *p5cs1* single mutants. Accordingly, mature pollen was collected from a homozygous *p5cs1* single mutant, and analyzed for pollen morphology and vitality by microscopic analysis, Alexander's stain, DAPI stain, and *in vitro* germination assay. As shown in Figure 9, microscopic and functional analysis of *p5cs1* pollen grains revealed the presence of misshaped (Figure 9A-D), non-viable (Figure 9D) and degenerated (Figure 9B and C) pollen grains, although to a lesser extent (~ 18%) compared to pollen from *p5cs1 p5cs2/P5CS2* mutants. Moreover, as shown in Additional file 2: Figure S2, pollens from *p5cs1* single mutants grown on germination medium, exhibited a ~ 12% reduction of germination rate, compared to wild type pollens. As seen in Additional file 2: Figure S2, A the percentage of germinated versus total pollen, including both large and small pollen grains, is 40 ± 2,0%, compared to 54 ± 2,9% of wild type control, confirming that male gametophytic defects are present, to a lesser extent, in *p5cs1* single mutants too. In addition, when large and small pollen grains are scored separately (Additional file 2: Figure S2, B), the percentage of large versus total large germinated pollen is 48 ± 2,5%, compared to 54 ± 2,9% of wild type control, while small pollen grains were never seen to germinate. As in *p5cs1* single mutant the percentage of large germinated pollen grains is not significantly different from that of wild type pollen grains, the 12% reduction of germination efficiency must be accounted for by the small and degenerated pollen fraction.

### Proline content analysis and exogenous proline treatment of pollen from *p5cs1 p5cs2/P5CS2* mutants provides a direct correlation between proline and pollen development

**Figure 11:**
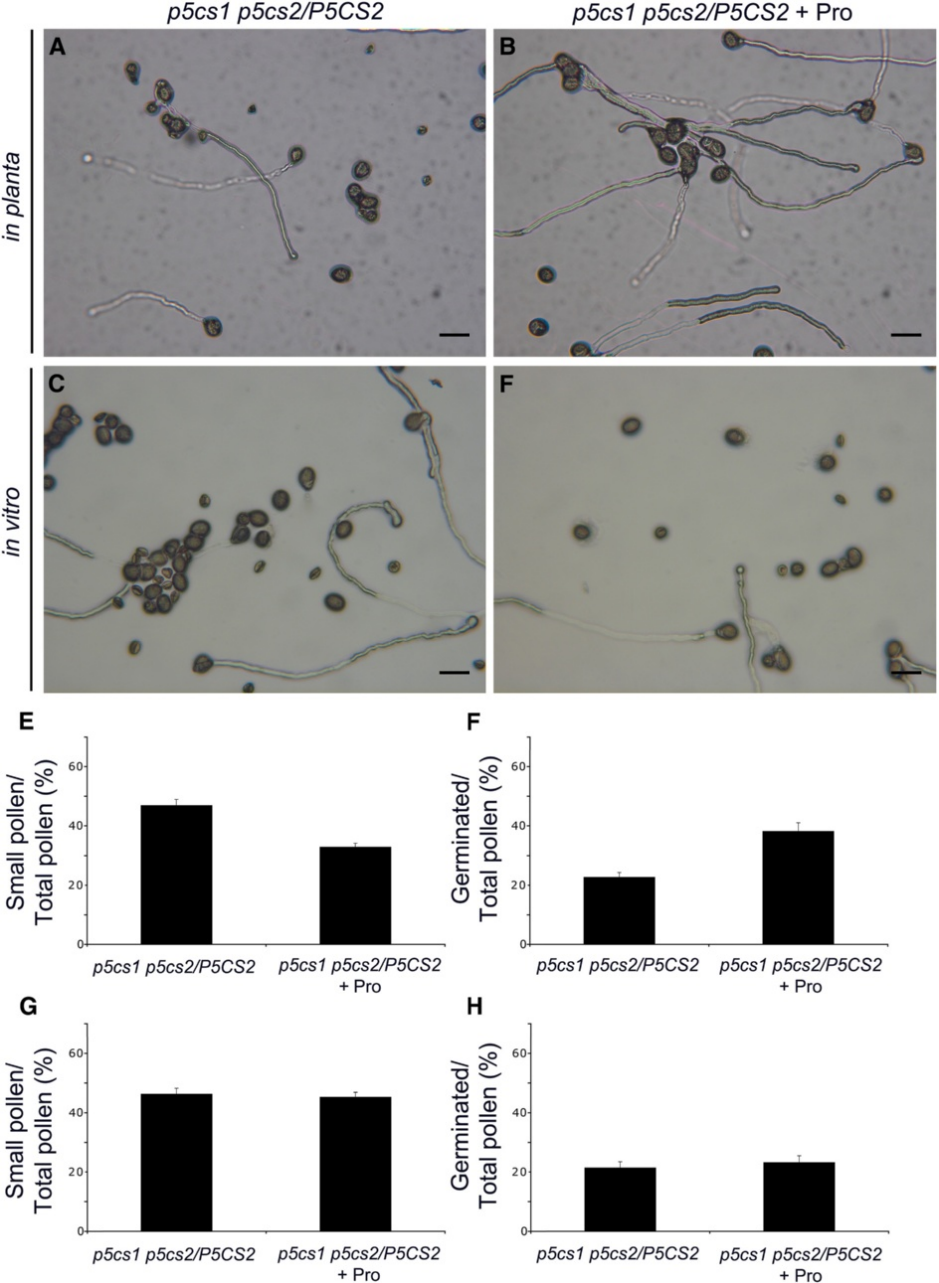
**Exogenous proline treatment of anthers and pollen from *p5cs1 p5cs2/P5CS2 *plants.** To confirm a proline requirement for pollen development and functionality 10 μM proline was supplied either *in vitro* to germinating pollens (**C**,**D**,**G**,**H**) or *in planta* to developing anthers (**A**,**B**,**E**,**F**). While *in vitro* proline treatment of pollen from *p5cs1 p5cs2/P5CS2* plants produces no differences in germination percentage (E, F), *in planta* supplementation gives rise to a significant improvement of germination efficiency (G, H). Bars = 50 μm. Values in (E to F) represent the means of four independent experiments ± SE.

On the whole, the data presented above point to a requirement for *p5CS1* and *p5CS2* in pollen development and functionality. To establish a direct correlation between proline and pollen development, we measured the proline content of pollen from *p5cs1 p5cs2/P5CS2* plants, compared to wild type. About 7.000 mature pollen grains from either wild type or *p5cs1 p5cs2/P5CS2* mutants were collected on a microscope slide and processed with a modification (see methods for details) of the Bates method [29]. The measurements were repeated in three independent experiments and expressed as average values ± SE. A clear difference in proline content was detected between the two pollen populations, as 336 ± 31 ng of free proline, roughly corresponding to 48 pg/pollen, was detected in wild type pollen, while only 105 ± 23 ng of free proline, corresponding to 15 pg/pollen of proline, could be detected in the mixed pollen population (large and small pollen grains) from *p5cs1 p5cs2/P5CS2* plants. The low content of proline measured in pollen from mutant plants (15 pg/pollen), compared to that found in wild type pollen (48 pg/pollen), provides a direct correlation between proline deficiency and pollen defects. Because in a pollen population from *p5cs1 p5cs2/P5CS2* plants, approx 50% of the pollen grains look aberrant, a ~ 50% reduction of proline content is expected. However, a proline reduction exceeding 70% was detected in pollen from mutant plants. This result suggests that the observed proline reduction cannot be accounted for only by the abortion of the small misshaped pollen, but that a reduction in proline content must take place, to some extent, also in the large wild type-looking pollen grains. In addition, 10 μM L-proline was supplemented *in vitro* to mature pollen from *p5cs1 p5cs2/P5CS2* plants grown on germination medium, or *in planta* to developing anthers. As shown in Figure 11, while no significant effect on pollen germination was produced by proline supplemented *in vitro* (Figure 11C, D, G and H), a significant complementation of pollen defect was observed when proline was sprayed daily to inflorescence buds, from early developmental stages to fully developed dehiscent anthers: the percentage of aberrant pollens decreased from 47.0 ± 2.5% to 33.0 ± 1.5% (Figure 11E), and the percentage of germinated pollens increased from 21.3 ± 1.3% to 39.2 ± 2.1% (Figure 11F). This indicates that exogenous proline can partially rescue the defects of pollen from *p5cs1 p5cs2/P5CS2* plants*,* when supplemented from the beginning of pollen development, confirming that proline plays a role in pollen development.

## Discussion

On the basis of crosses showing that pollen with genetic defects in both *P5CS1* and *P5CS2* is almost completely unable of a successful fertilization, a requirement for proline in male gametophyte viability was hypothesized and demonstrated here, by means of genetic, developmental and molecular evidence.

### Proline is required for pollen development and fertility

Massive accumulation of proline has been reported in anthers and pollen by different authors in a number of species [7-11,14], but it is not yet clear why such high amount of proline is required. Functions as diverse as free radical scavenger [12], protector of membranes and cellular structures [13], energy source [15], metabolic precursor [14], and main amino acid constituent of hydroxyproline-rich cell walls [16] have been proposed but none of these possibilities has attained wide acceptance, and the interesting hypothesis that multiple functions may be accounted for by proline action has been suggested [30].

Evidence provided in this work indicates that pollen from mutants homozygous for *p5cs1* and heterozygous *for p5cs2* can transmit the *p5cs2* mutation with an overall frequency of about 0.8%, and that mutants with decreasing levels in proline content [2,3,24] have increasing problems in pollen viability indicating that proline is required for male fertility.

### Proline accumulation in pollen may rely on endogenous proline synthesis

Apart from the defects in pollen development described in this work, and from a delay in flower transition described by Mattioli et al. [1], the vegetative and reproductive growth of mutants homozygous for *p5cs1* and heterozygous for *p5cs2,* including the development of the female gametophyte, is essentially normal. This evidence implies that the small amount of proline coming from the activity of the wild type *P5CS2* allele, always present in the sporophytic tissues of *p5cs1 p5cs2/P5CS2*, is sufficient for normal growth and reproduction, but not for proper pollen development.

The specific requirement of proline for pollen development and function, is confirmed by the high amount of proline found in pollen by different authors [7-11,14], by the very low level of proline measured in mutant pollen, and by the partial rescue of pollen defects obtained by exogenous proline treatment. While this complementation provides direct proof that proline is required for pollen development and function, it gives no indication whether the required proline derives uniquely from endogenous synthesis inside the pollen or also from proline synthesized in nearby mother cells and transported or diffused inside pollen grains. This point clearly needs to be understood in future works.

However, since pollen from *p5cs1 p5cs2/P5CS2* is infertile, a possible transport or diffusion of proline synthesized in surrounding sporophytic cells by the residual *P5CS2* allele is obviously insufficient. Furthermore, single, double and triple knockout mutants of the *AtProT* genes (At2G39890, At3G55740, At2G36590) responsible for proline transport in plants, have been isolated and characterized but none of them revealed alterations, compared to wild type, neither in proline content nor in pollen germination efficiency [19]. Although the possibility that different carriers, such as AtLHT5 (At1g67640) [31] or AtLHT7 (At4G35180) [32], may compensate, overlap to, or substitute for AtProTs, current evidence does not support a role of transport for proline accumulation in pollen grains. In addition, microarray data indicate that all the genes involved in proline synthesis are strongly expressed in pollen [33,34], and data from Szekely et al. [24], who detected in the pollen of Arabidopsis the expression of both AtP5CS1–GFP and AtP5CS2–GFP, confirm the presence of the P5CS protein in the male gametophyte. Overall these data suggest that proline is actively synthesized in pollen.

The question whether proline may be synthesized directly in pollen grains has been the object of controversial discussions, because some authors could detect low [8,32] or no expression of *P5CS* in pollen [19], while others reported the presence of an AtP5CS-GFP protein in the pollen of Arabidopsis, suggesting that biosynthesis of proline takes place in this organ [24]. However, the discrepancy in the expression levels of *P5CS* gene as observed by different authors [8,19,24,32], may depend on the developmental stage in which pollen was analyzed, and we may speculate that *P5CS1/2* genes could be expressed only in particular stages of pollen development, and still accumulate enough P5CS enzyme to satisfy overall proline demand for pollen maturation. Temporal discrepancies between transcript and protein levels have been reported in pollen also for other genes, such as *AtSUC1* (AT1G71880), whose transcript level is high at tricellular stage and low in mature pollen [35] and *AtSTP9* (AT1G50310), whose gene product can only be detected by immunofluorescence microscopy after the onset of germination [36]. In addition, as above stated, high levels of expression of either *P5CS1*, *P5CS2*, and *P5CR* are detected in pollen by microarray analysis [33,34], directly confirming that endogenous proline synthesis from glutamate takes place in pollen grains. Unexpectedly, microarrays analysis also detects the expression of δ-OAT in pollen, although ornithine pathway seems not able to compensate P5CS deficiency in pollen from *p5cs1 p5cs2/P5CS2*. These contrasting pieces of evidence can be reconciled if ornithine pathway does not contribute to proline synthesis. Incidentally, this evidence supports the finding of Funck et al. [22] who demonstrated that the ornithine pathway is essential for arginine catabolism but not for proline synthesis.

Overall, proline accumulation in pollen may rely essentially on endogenous proline synthesis, although is yet to be understood whether proline derives uniquely from endogenous synthesis inside the male gametophyte or also from proline synthesized in nearby sporophytic cells and transported or diffused inside pollen grains. A likely hypothesis is that, as pollen lose desmosomal connections to surrounding sporophytic cells, becomes dependent on endogenous proline synthesis, consistent with the late appearance (stage 11) of visible aberrations in developing pollen from *p5cs1 p5cs2/P5CS2*, and with the absence of obvious defects in female gametopytes, always embedded in sporophytic cells.

### Relationship between *p5cs* and *p5cr* mutants

Intriguingly, two Arabidopsis mutants bearing T-DNA insertions on *P5CR*, *emb-2772-1* and *emb-2772-2*[37] exhibit an embryo lethal phenotype, similarly to *p5cs2* mutants, halting embryo development at a preglobular stage. In sharp contrast, however, no gametopytic defects have been associated, so far, to these mutants.

While it is not surprising that lesions in *P5CR* and *P5CS2*, two genes coding for proline synthesis enzymes belonging to the same pathway, may lead to similar defects in embryo development, it is puzzling that, contrary to *p5cs2*, *emb-2772* exhibits no gametophytic defects. In Arabidopsis a number of mutants have been described by Muralla et al. [38] with defects in embryo but not in gametophyte development.

To explain this apparent paradox, the authors propose that gene products derived from transcription of wild type alleles in heterozygous sporocytes may compensate the deficiency of the mutant gametophytes, and that embryo lethality results when these products are eventually depleted. Likewise, we may speculate that, contrary to P5CS2, the P5CR transcript and/or protein, synthesized in heterozygous sporocytes, is stable enough to sustain pollen but not embryo development.

### Proline may have distinct roles in pollen development and germination

The data presented here suggest that proline is required for pollen development, but gives no indication on the role of proline in pollen development. We know from histological analysis (Figure 5) that a fraction of pollen grains begins to look shriveled and shrunk from stage 11, when, after completion of the two mitotic divisions, the microspores start their maturation to pollen grains. As pollen development proceeds, it becomes more and more desiccated, and increasing amounts of proline may be needed to avoid protein denaturation and preserve cellular structures, including nuclei, as hypothesized by Chiang and Dandekar [7]. A role for proline in the protection of cellular structures from denaturation has been proposed by different authors either as compatible osmolyte [7], scavenger of free radicals [12,24], or as protector of membranes and cellular structures [13]. Although, from available data, a defect in mitotic divisions cannot be ruled out, the degeneration of the cellular structures observed in pollen grains of *p5cs1 p5cs2* genotype, may be caused by the irreversible damages on cellular membranes caused by the process of dehydration in absence of the protective action of proline.

Once pollen has reached full maturation, accumulated proline is catabolized and serves as source of energy - to fuel the rapid and energy-demanding elongation of the pollen tube [15,39] - and/or as metabolic precursor of γ-amino butyric acid (GABA), the catabolism of which has been shown essential for late pollen tube elongation and guidance [40].

In the future it will be interesting to address this issue by uncoupling these two putative functions, for example targeting in developing pollen grains from *p5cs1 p5cs2/P5CS2* plants non-metabolizable compatible osmolyte, such as glycine betaine. Equally interesting it will be to dissect the role of proline synthesized in the haploid male gametophyte from that synthesized in diploid sporophytic tissues of the anther.

## Conclusions

We show here that in mutants homozygous for *p5cs1* and heterozygous *for p5cs2,* defective in proline synthesis, the development of the male gametophyte with mutations in both *P5CS1* and *P5CS2* is severely compromised, and provide genetic evidence that proline is needed for pollen development and fertility.

## Methods

### Plant growth conditions, segregation and embryo analyses

Wild-type and mutant *Arabidopsis thaliana* from Columbia-0 (Col-0) ecotype used in this work were grown in a growth chamber at 24/21°C with light intensity of 300-μE·m^-2^·s^-1^ under 16 h light and 8 h dark per day. Arabidopsis homozygous for *p5cs1* (SALK_063517), originally obtained from the SALK collection, are knockout insertional mutants described in [3] containing a pROCK-derived T-DNA within exon 14. Arabidopsis heterozygous for *p5cs2* (GABI_452G01)*,* originally obtained from the GABI-Kat collection, are insertional mutants described in [1], containing a PAC161- derived T-DNA within exon 18. As reported in [1,3]*p5cs2/P5CS2* is embryo lethal in homozygous state and must be propagated in heterozygous state*.* Arabidopsis homozygous for *p5cs1* and heterozygous for *p5cs2* (*p5cs1 p5cs2/P5CS2*)*,* have been characterized and described elsewhere [1,3]. For segregation analysis, seeds from a self-fertilized *p5cs1 p5cs2/P5CS2* plant were stratified for three days at 4°C, surface-sterilized, and germinated on MS_1/2_ plates supplemented with 12 μg/ml sulfadiazine. Segregation ratios were calculated by scoring the number of resistant over susceptible plantlets, and confirmed by PCR analysis of random samples. using primers 5’-CAAGCAATGGTGGAAGAGTAAA-3’ and 5’- CGGGGCTCAAGAAAAATCC -3’ for the sulfadiazine resistance gene. For embryo analysis, siliques derived from self-fertilized wild types, *p5cs2*/P5CS2 mutants, or *p5cs1 p5cs2*/*P5CS2* mutants, were dissected and analyzed under a Zeiss Stevi SV 6 light stereomicroscope (Carl Zeiss Microimaging GmbH, Jena, Germany). Digital images were acquired with a Jenoptik ProgRes® C3 digital camera (Jenoptik, Jena, Germany). All the analyses have been repeated at least four times. Statistical significance was inferred from percentage data by using χ^2^ analysis.

### Plant crosses

In crosses between *p5cs1 p5cs2/P5CS2* and flowering time mutants the F1 generation was allowed to self-fertilize and the presence of the *p5cs2* mutant allele was assessed from the F2 generation, by sulfadiazine selection or by PCR genotyping of the sulfadiazine resistance gene. To confirm the data and rule out any possible interference of the flowering time mutant genotypes in the transmission of the *p5cs2* mutation, reciprocal crosses between *p5cs1 p5cs2/P5CS2* and wild type were performed. Transmission of the T-DNA insertion on *P5CS2* gene was assessed, either by sulfadiazine selection of outcrossed seeds germinated on sulfadiazine-containing solid medium, or by PCR genotyping of sulfadiazine resistance gene on plantlets grown without selection Statistical significance was inferred from percentage data by using χ^2^ analysis.

### Morphological and functional pollen characterization

For orcein staining, pollen was collected by dabbing mature flowers, from four weeks old plants, on a microscope slide. After a brief incubation in 1% acetic orcein, the pollen grains were rinsed in 50% acetic acid and examined under a Leitz Laborlux D light microscope (Leitz, Wetzlar, Germany) equipped with a Jenoptik ProgRes® C3 digital camera (Jenoptik, Jena, Germany). For histological analysis, floral buds, of different developmental stages, were embedded in Technovit 7100 (Kulzer), and 3-mm cross-sections were stained with 1% Toluidine blue as described in [41] and analyzed under a Leitz Laborlux D light microscope (Leitz, Wetzlar, Germany). For evaluation of pollen vitality, flower buds or isolated anthers were collected, fixed overnight in Carnoy’s fixative (6 alcohol:3 chloroform:1 acetic acid), and stained with a modified Alexander’s stain as described by Peterson et al. (2010). For DAPI (4’, 6-diamidino-2-phenylindole) staining [42], mature pollen was collected, incubated 30’ in DAPI staining solution (0.1 M sodium phosphate buffer, pH 7, 1 mM EDTA, 0.1% Triton-X-100 and 0.4 μg/ml DAPI) and analyzed with a Zeiss Axioskop 2 plus microscope (Carl Zeiss Microimaging GmbH, Jena, Germany) equipped with a DAPI filter set consisting of an excitation filter (BP 365/12 nm), a beam splitter (395 nm), and an emission filter (LP 397 nm). Acquisition of digital images was made with a Jenoptik ProgRes® C3 digital camera (Jenoptik, Jena, Germany).

### Molecular analysis

Pollen grains were separated by size (large and small) under a Zeiss Stevi SV 6 light stereomicroscope (Carl Zeiss Microimaging GmbH, Jena, Germany) and pools of about 500 pollen grains were prepared and frozen at −20°C. Pollen DNA was extracted from these samples with a modified CTAB (cetyl trimethylammonium bromide) protocol according to [43]. Because of the presence of a tough pollen coat, major modifications were introduced in the initial steps consisting in squashing pollens between a microscope slide and a cover slip, retrieving them with 50 μl CTAB buffer and heating the solution for 15’ at 95°C. PCR conditions were 3’ at 94°C followed by 35 cycles of 30” at 94°C, 1’ at 58°C, and 50” at 72°C. The primer pairs used were 5’-CAAGCAATGGTGGAAGAGTAAA-3’ and 5’-CGGGGCTCAAGAAAAATCC-3’ for the sulfadiazine resistance gene, 5’-GGAGCAGAATGGTTTTCTCG-3’ and 5’-TATCTGGGAATGGCGAAATC-3’ for the T-DNA insertion on *P5CS2*, 5’-GGAGCAGAATGGTTTTCTCG-3’ and 5’-TGGAAAACAGCAGCACTGTC - 3’ for the gene *P5CS2,* 5’-CTGTTGGGGGTAAACTCATTG-3’ and 5 -GCGTGGACCGCTTGCTGCAACT-3’ for the T-DNA insertion on *P5CS1,* 5’-CTGTTGGGGGTAAACTCATTG-3’ and 5’-CTCTGCAACTTCGTGATCCTC-3’ for the gene *P5CS1.*

### *In vitro* pollen germination

Mature pollens from stage 13 flowers [44] were collected, transferred to glass slides coated with freshly prepared germination medium (5 mM CaCl2, 1 mM MgSO4, 5 mM KCl, 0.01 mM H3BO3, 10% sucrose and 1.5% agarose, pH 8.0), and kept overnight in a moist chamber at 21°C. To minimize *in vitro* germination variability, pollen from wild type plants was always included nearby pollen from *p5cs1 p5cs2/P5CS2* plants on the same microscope slide.

For pollen germination analysis, the slides were examined under a Leitz Laborlux D microscope (Leitz, Wetzlar, Germany) and digital pictures of randomly chosen fields were acquired with a Jenoptik ProgRes® C3 digital camera (Jenoptik, Jena, Germany). To determine pollen germination efficiency, the number of germinated and non-germinated pollens was scored from five, randomly chosen, fields per replica in four independent experiments. Statistical significance was inferred from percentage data by using χ^2^ analysis.

### Proline determination and exogenous proline complementation

Proline measurements were modified from [29] as follows: Mature pollen grains (~ 5–10.000) were collected on a microscope slide, smashed with a slide cover glass, and retrieved with 30 μl of a 3% (w/v) aqueous solution of sulfosalicylic acid. After centrifugation (12.000 ×g for 10’), the supernatant was added to 30 μl of acid-ninydrin and 30 μl of glacial acetic acid and let react for 2 hours at 80°C in a micro-test tube made up by the tip of pasteur pipette heat-sealed at the two ends. The reaction mixture was extracted with 60 μl of toluene and its optical density measured at 520 nm with a NanoDrop 2000 micro-spectrophotometer (Thermo Scientific, Wilmington, USA). Proline concentration was determined from a standard curve built with L-proline. *In vitro* complementation was attempted by spotting mature pollens in microscope slides coated with germination medium supplemented with 10 μM L-proline. *In vivo* complementation was performed by daily spraying early developing inflorescences with 10 μM proline. Mature pollens from stage 13 proline-treated flowers were collected and analyzed as described above.

## Competing interests

The authors declare that they have no competing interests.

## Authors’ contributions

MT wrote and supervised the work, performed reciprocal crosses, orcein stains and Alexander’s stains, RM carried out segregation and silique analyses, DAPI stains, germination assays and proline complementation assays, CL carried out histological analyses, MB performed molecular analyses, PC gave financial support and revised the manuscript. All authors read and approved the final manuscript.

## Supplementary Material

Additional file 1 **Figure S1. ***p5cs1 p5cs2/P5CS2* anther stained with Alexander’s stain. Close up of a *p5cs1 p5cs2/P5CS2* anther stained with Alexander’s stain. The small and misshaped pollen grains are clearly visible, within a *p5cs1 p5cs2/P5CS2* anther, as non-stained pollen pollen grains alongside wild type-like, red stained, pollen grains. Bar = 50 μm. Click here for file

Additional file 2 **Figure S2. ***In vitro* germination assays of pollen from *p5cs1* single mutants. To evaluate possible defects in pollen viability of a mutant bearing a single mutation in *P5CS1* gene, pollen from a homozygous knockout *p5cs1* mutant was incubated *in vitro* on germination medium and scored for germination. (A) Percentage of germinated versus total pollens (germinated + non germinated), including both large and small pollen grains (right column), compared to wild type (left column). (B) Percentage of large (middle column), small (right column), and wild type pollen (left column) versus large (middle column), small (right column) and wild type (left column) total germinated pollen. Values in (A) and (B) represent the means of four independent experiments ± SE. Click here for file
