# Supplementary figures and images for: Proline is required for male gametophyte development in Arabidopsis
Source: BMC Plant Biol. 2012 Dec 12;12:236. doi: 10.1186/1471-2229-12-236 (PMC3543202; doi:10.1186/1471-2229-12-236)

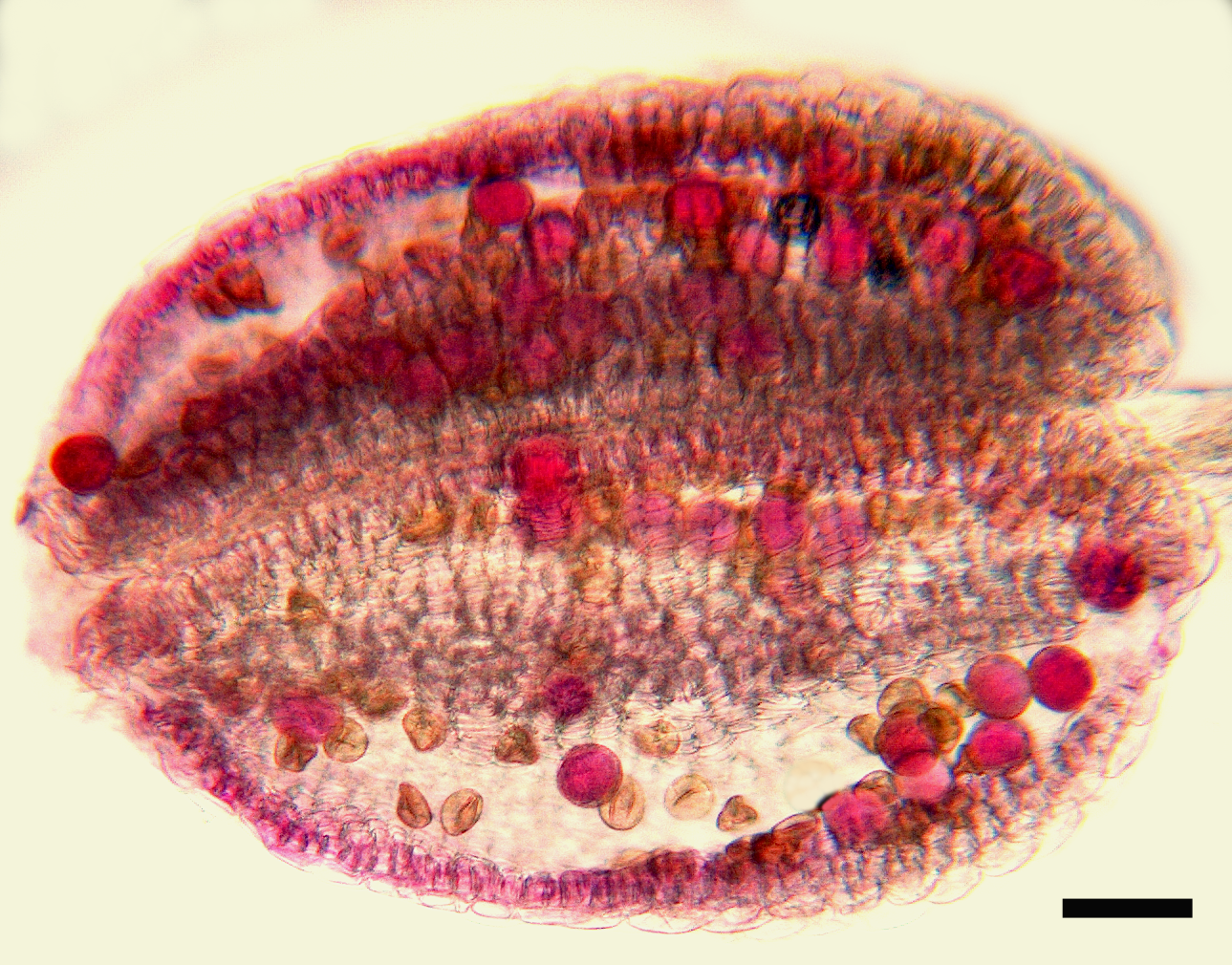

Supplement: Additional file 1 — Figure S1. p5cs1 p5cs2/P5CS2 anther stained with Alexander’s stain. Close up of a p5cs1 p5cs2/P5CS2 anther stained with Alexander’s stain. The small and misshaped pollen grains are clearly visible, within a p5cs1 p5cs2/P5CS2 anther, as non-stained pollen pollen grains alongside wild type-like, red stained, pollen grains. Bar = 50 μm. [file 1471-2229-12-236-S1.tiff]

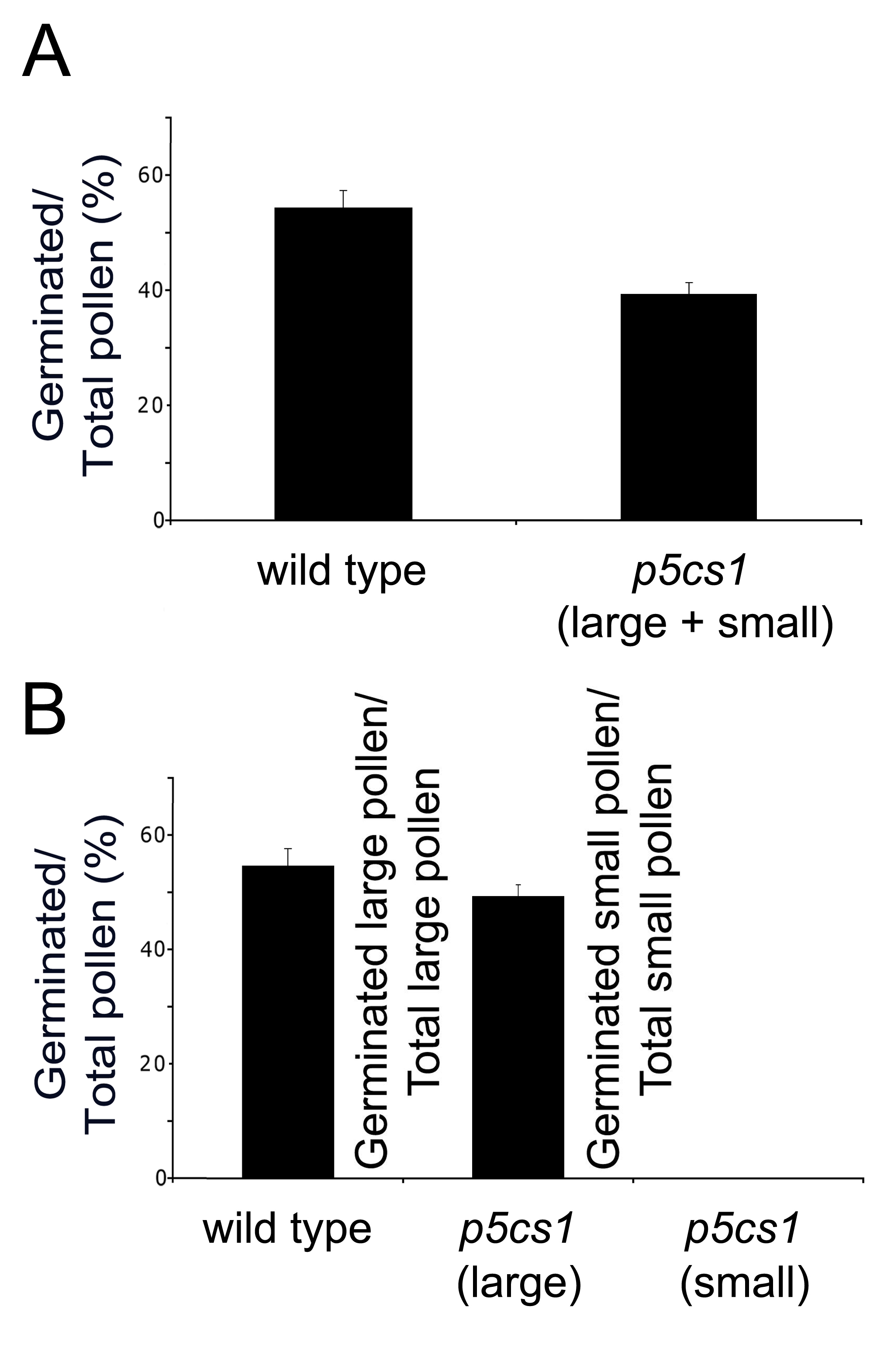

Supplement: Additional file 2 — Figure S2. In vitro germination assays of pollen from p5cs1 single mutants. To evaluate possible defects in pollen viability of a mutant bearing a single mutation in P5CS1 gene, pollen from a homozygous knockout p5cs1 mutant was incubated in vitro on germination medium and scored for germination. (A) Percentage of germinated versus total pollens (germinated + non germinated), including both large and small pollen grains (right column), compared to wild type (left column). (B) Percentage of large (middle column), small (right column), and wild type pollen (left column) versus large (middle column), small (right column) and wild type (left column) total germinated pollen. Values in (A) and (B) represent the means of four independent experiments ± SE. [file 1471-2229-12-236-S2.jpeg]
